# Impact of degenerative disc changes on the early outcomes in patients undergoing full-endoscopic lumbar discectomy—a prospective study

**DOI:** 10.1097/j.pbj.0000000000000313

**Published:** 2025-12-17

**Authors:** Pedro Valente Aguiar, Diogo Bonifácio Afonso, Paulo Miguel da Silva Pereira, Pedro Santos Silva

**Affiliations:** a Faculty of Medicine, University of Porto, Porto, Portugal; b Department of Neurosurgery, Unidade Local de Saúde de São João, EPE, Porto, Portugal

**Keywords:** lumbar disc herniation, endoscopic lumbar discectomy, degenerative disc disease, Pfirrmann grading system, low back pain

## Abstract

**Introduction:**

Lumbar disc herniation (LDH) is a prevalent condition that can cause radiculopathy and leg pain. Full-endoscopic lumbar discectomy (FELD) has emerged as a minimally invasive alternative to microdiscectomy, offering similar clinical efficacy with potentially reduced recovery times. Degenerative disc disease (DDD) is frequently observed in patients undergoing surgery, yet its influence on surgical early outcomes of FELD remains unclear.

**Methods:**

This prospective study included 91 patients who underwent FELD for single-level LDH between January 2022 and March 2024. Patients were classified in two groups based on Pfirrmann grade: grade inferior to 4 (Group 1) and grade 4 or 5 (Group 2). Clinical outcomes were assessed using the Numeric Rating Scale (NRS) for back and leg pain preoperatively and at 2, 14, 30, and 90 days postoperatively, and the Oswestry Disability Index (ODI) preoperatively and at 30 and 90 days postoperatively.

**Results:**

Among the 91 patients, 89% had advanced disc degeneration (Pfirrmann grade 4 or 5). Preoperatively, Group 1 (all Pfirrmann 3) had significantly higher back pain (NRS 8.2 vs. 5.2, *P* < 0.01), while Group 2 had a greater difference between leg and back pain (*P* = 0.041). Postoperatively, both groups experienced significant pain relief, but Group 1 showed superior back pain improvement across all time points. No significant differences were observed in ODI scores or leg pain improvement.

**Discussion:**

Patients with moderate disc degeneration (Pfirrmann grade 3) exhibited greater preoperative back pain but experienced superior back pain relief after FELD compared with those with advanced degeneration (Pfirrmann grade 4 or 5).

## Introduction

Lumbar disc herniation (LDH) can lead to leg pain and radiculopathy due to nerve root compression^1^ and is a common reason for seeking medical aid. Pojskic et al^2^ suggested that the risk of developing LDH is about 30% during one's lifetime. As for symptomatic disc herniation, the risk ranges from 1 to 3%. Of these, about 60%–90% resolve spontaneously, while the remaining may need surgical treatment.

In cases of persistent radiculopathy or leg pain, discectomy is an efficient treatment option^3,4^. Full-endoscopic lumbar discectomy (FELD) has emerged as an alternative to microdiscectomy with similar clinical results but shorter hospitalization times and less intraoperative blood loss^5,6^.

Spine and intervertebral disc (IVD) degeneration result from abnormal, cell-mediated responses leading to progressive structural failure^7^. Degenerative disc disease (DDD) has been associated with symptoms such as low back pain (LBP)^8^; however, Brinjikji W, et al^9^ demonstrated that DDD is also very common in asymptomatic adults, especially as age progresses.

Whether lower back radiological changes are relevant in predicting future or to justify current lower back pain, there is evidence suggesting that these changes may worsen the clinical outcome of spinal discectomy^10^. Recently, Papanastasiou et al^11^ showed that DDD on the operated and adjacent discs was associated with worse clinical outcomes up to 5 years postoperatively after microdiscectomy.

DDD has been evaluated with imaging methods such as MRI. The Pfirrmann grading system^12^ is a widely used classification for assessing intervertebral disc degeneration based on T2-weighted MRI scans. Modic Changes (MC) classification^13^ describes pathological alterations in the vertebral endplates and adjacent bone marrow, seen on MRI using T1-weighted and T2-weighted images.

To the best of our knowledge, no previous studies have studied the influence of degenerative disease on the early clinical outcomes of patients undergoing FELD. With the objective of providing the best information for decision making before lumbar herniation surgery, this study aims to investigate possible relations between radiological evidence of spinal degeneration and early surgery outcomes.

## Methods

### Trial design, participants, and setting

This prospective study was approved by the ethics committee of our institution and followed the STROBE^14^ statement for observational studies.

The study population consisted of adult patients with LDH and degenerative lumbar disease who underwent endoscopic lumbar discectomy between January 2022 and March 2024 due to single level lumbar disc herniation.

This study took place in a tertiary Portuguese neurosurgical center, and lumbar discectomy was indicated when patients presented with lumbar radiculopathy or leg pain refractory to best conservative treatment for at least 8 weeks due to single level disc herniation.

Exclusion criteria included intraoperative dural tear, multilevel surgery, or surgical approaches wherein lumbar discectomy was not the main decompressive procedure.

### Clinical data

Patients'  clinical characteristics including age, gender, surgical level, main clinical presentation, symptom duration, type of endoscopic approach, and complications were collected both from the clinical registries at our institution and the EUROSPINE Spine Tango registry.

To evaluate the impact of degenerative disc disease on the early clinical outcomes of patients submitted to lumbar discectomy, patients were asked to fulfil a Numeric Rating Scale (NRS) for back and leg pain and the Oswestry Disability Index (ODI) before surgery. The NRS ranged from 0 to 10 in which 0 represents no pain and 10 represents the highest possible.

Back pain and leg pain were reassessed by phone at 2 and 14 days after surgery through the NRS, while patients fulfilled the back and leg NRS and ODI 30 days after surgery in the outpatient clinic. Ninety days after the procedure phone calls were conducted to assess the following variables: leg and back NRS, ODI, complications, return to work, and pain medication.

### Surgical procedure

A spine surgeon with experience in FELD performed all procedures under general anesthesia at the ambulatory center of our institution. An interlaminar or transforaminal approach was used according to level, characteristics of disc herniation, and degenerative pathology.

### Intervertebral disc degeneration assessment

Pfirrmann Grading System and Modic Changes were assessed using lumbar spine MRIs performed during the preoperative study of each patient. T2-weighted images were used to classify intervertebral disc changes according to the Pfirrmann et al^12^ grading system. This tool categorizes disc degeneration into 5 grades (I‒V) by evaluating disc structure, signal intensity, and nucleus pulposus homogeneity. As grades progress signal intensity diminishes, and the disc structure becomes increasingly irregular, ultimately leading to collapse and deformation. Pfirrmann grading system was applied to the intervertebral disc associated with the lesion, as well as both the intervertebral discs one level above and one level below (except when L5-S1 level was operated on). Modic changes^13^ were classified based on T1-weighted and T2-weighted images, with focus on vertebral body alterations adjacent to the IVD operated. It ranges from MC 1 to MC 3, and the progression reflects a shift from early inflammatory changes with edema to fatty degeneration of the bone marrow and, ultimately, to advanced sclerosis. This evolution relates to increasing structural deterioration and reduced regenerative potential of the affected vertebral segments.

### Outcome

The primary objective of the study was to assess whether advanced DDD defined by Pfirrmann grade 4 or 5 led to differences in mean improvement in either leg or back NRS during the analyzed timeframes in comparison to Pfirrmann grade 3 or lower. Secondary objectives included the impact of Pfirrmann grade 4 or 5 on ODI and the impact of Modic changes on patient outcomes (NRS and ODI).

### Statistical analysis

R software (R Foundation for Statistical Computing, Vienna, Austria) version 4.0.3 was used for data analysis.

Kruskal-Wallis (KW) tests were used for median comparison between independent samples, and the Fisher test was used for associations between categorical variables. Two-way mixed analysis of variance (ANOVA) was used to evaluate the effect of a factor in a continuous outcome across time, and post hoc tests were performed using Bonferroni correction.

## Results

Ninety-one patients (57 women, 34 men) were evaluated with a mean age of 44.7 years (SD 11.8). Two groups were analyzed based on the level of disc degeneration, specifically on the operated level; group 1 included 10 patients with Pfirrmann grade 3, while group 2 had 81 patients with either Pfirrmann grade 4 or 5.

Symptom duration was comparable between groups (*P* = 0.792), with most patients experiencing symptoms for more than 6 months (65%) before surgery, while 20% had symptoms for 3–6 months, and 15% experienced symptoms for less than 3 months (Table 1 presents patient characteristics).

**Table 1:** Patient characteristics, total, and according to Pfirrmann

	Population	Pfirrmann 3	Pfirrmann 4 or 5	*P*
Total	91	10	81	
Age				0.75*
Mean (SD)	44.7 (11.8)	43.0 (10.2)	45.0 (12.1)	
Sex				0.04†
Female	57 (63%)	3 (30%)	54 (67%)	
Male	34 (37%)	7 (70%)	27 (33%)	
Level				1.00†
L3‒L4	2 (2%)	0 (0%)	2 (2%)	
L4‒L5	42 (46%)	5 (50%)	37 (46%)	
L5‒S1	34 (37%)	5 (50%)	42 (52%)	
Endoscopic Approach				0.72†
Interlaminar	61 (67%)	6 (60%)	55 (68%)	
Transforaminal	30 (33%)	4 (40%)	26 (32%)	
Symptoms duration				0.79†
<3 months	14 (15%)	1 (10%)	13 (16%)	
3–6 months	18 (20%)	1 (10%)	17 (21%)	
>6 months	59 (65%)	8 (80%)	51 (63%)	
Previous surgery				0.59†
No	81 (89%)	10 (100%)	71 (88%)	
Yes	10 (11%)	0 (0%)	10 (12%)	

* Kruskal-Wallis rank-sum test.

† Fisher exact test for count data.

**Figure 1.**
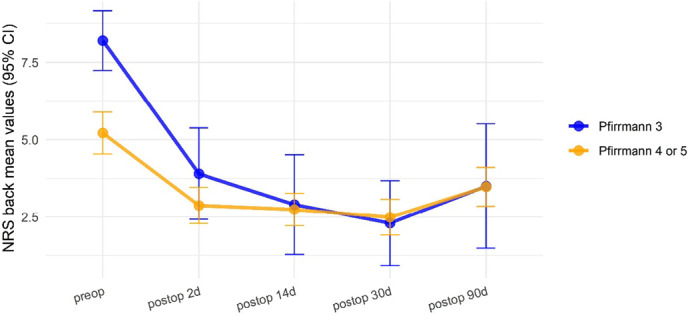
Back NRS scores by Pfirrmann grade of operated disc.

The mean preoperative back pain as measured with NRS was 8.2 (SD: 1.5) in group 1 and 5.2 (SD: 3.1) in group 2. Regarding back pain across time, a significant interaction between Pfirrmann group and time (*P* = 0.02, mixed ANOVA) was observed, with a significant difference of back NRS between groups at preoperative time (*P* = 0.004, post hoc test adjusted with Bonferroni), but with no differences in back pain between groups in postoperative period (Fig. 1). No differences were found between groups regarding preoperative leg pain, ODI, surgical level, type of approach, and Modic changes.

**Figure 2.**
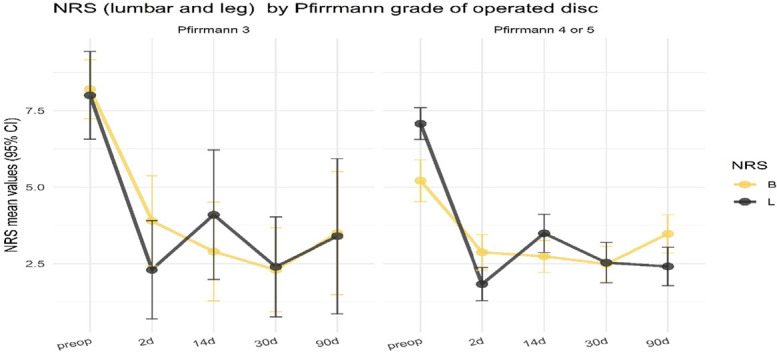
Back and leg NRS, through the different timeframes, by Pfirrmann grade of operated disc.

Preoperatively, a distinction between the two groups was observed in the pain dominance, with the Pfirrmann 4 or 5 group reporting higher leg pain compared with back pain (mean difference 2.6 vs. 1.0; *P* = 0.041, KW) (Fig. 2).

Postoperatively both groups improved to similar NRS back and leg values; however, patients in group 1 experienced a greater mean improvement in back NRS than group 2 patients at all timeframes (Table 2 and Fig. 1). After surgery, there were no significant differences between groups in mean NRS leg, ODI improvement, and difference between leg and back pain.

**Table 2:** Patient outcomes, Pfirrmann grade, and Modic changes

	Pfirrmann 3 (N = 10)	Pfirrmann 4 or 5 (N = 81)	Total (N = 91)	*P*
Pfirrmann at operated level				<0.01*
Mean (SD)	3.0 (0.0)	4.5 (0.5)	4.3 (0.7)	
N-Miss	0	0	0	
Pfirrmann at the superior adjacent level				0.02*
Mean (SD)	2.4 (0.8)	3.2 (1.1)	3.2 (1.1)	
N-Miss	0	0	0	
Pfirrmann at the inferior adjacent level				0.69*
Mean (SD)	3.2 (1.3)	3.4 (1.3)	3.4 (1.3)	
N-Miss	5	42	47	
Modic changes type—superior vertebra				0.49†
0	4 (40%)	33 (41%)	37 (41%)	
1	1 (10%)	6 (7%)	7 (8%)	
2	4 (40%)	40 (49%)	44 (48%)	
3	1 (10%)	2 (2%)	3 (3%)	
N-Miss	0	0	0	
Modic changes type—inferior vertebra				0.70†
0	5 (50%)	37 (46%)	42 (46%)	
1	0 (0%)	3 (4%)	3 (3%)	
2	4 (40%)	38 (47%)	42 (46%)	
3	1 (10%)	3 (4%)	4 (4%)	
N-Miss	0	0	0	
Preoperative NRS back				<0.01*
Mean (SD)	8.2 (1.5)	5.2 (3.1)	5.5 (3.1)	
N-Miss	0	1	1	
Preoperative NRS leg				0.22*
Mean (SD)	8.0 (2.3)	7.1 (2.4)	7.2 (2.4)	
N-Miss	0	1	1	
Preoperative ODI				0.58*
Mean (SD)	51.2 (20.7)	47.3 (16.7)	47.8 (17.1)	
N-Miss	0	5	5	
ODI improvement 30 days postoperative				0.57*
Mean (SD)	27.3 (8.4)	32.8 (22.5)	32.3 (21.6)	
N-Miss	3	18	21	
ODI improvement 90 days postoperative				0.53*
Mean (SD)	35.6 (22.3)	28.7 (23.8)	29.4 (23.6)	
N-Miss	2	8	10	
NRS back improvement 2 days postoperative				0.04*
Mean (SD)	4.3 (2.1)	2.3 (3.7)	2.5 (3.6)	
N-Miss	0	3	3	
NRS back improvement 14 days postoperative				<0.01*
Mean (SD)	5.3 (2.5)	2.5 (3.3)	2.8 (3.3)	
N-Miss	0	1	1	
NRS back improvement 30 days postoperative				<0.01*
Mean (SD)	5.9 (2.6)	2.7 (3.5)	3.1 (3.5)	
N-Miss	0	11	11	
NRS back improvement 90 days postoperative				0.02*
Mean (SD)	4.7 (3.2)	1.7 (3.8)	2.1 (3.8)	
N-Miss	0	4	4	
NRS leg improvement 2 days postoperative				0.85*
Mean (SD)	5.7 (2.7)	5.3 (3.5)	5.3 (3.4)	
N-Miss	0	3	3	
NRS leg improvement 14 days postoperative				0.73*
Mean (SD)	3.9 (2.6)	3.6 (3.4)	3.6 (3.3)	
N-Miss	0	1	1	
NRS leg improvement 30 days postoperative				0.44*
Mean (SD)	5.6 (3.1)	4.6 (3.5)	4.7 (3.4)	
N-Miss	0	11	11	
NRS leg improvement 90 days postoperative				0.82*
Mean (SD)	4.6 (3.5)	4.7 (3.6)	4.7 (3.6)	
N-Miss	0	4	4	
Difference between leg and back NRS—preoperative				0.04*
Mean (SD)	1.0 (1.1)	2.6 (2.4)	2.4 (2.4)	
N-Miss	0	1	1	

* Kruskal-Wallis rank-sum test.

† Fisher exact test for count data.

For Modic changes at the superior vertebra, 41% of patients exhibited no Modic changes (Type 0), 8% had Type 1, 48% had Type 2, and 3% had Type 3. Similarly, at the inferior vertebra, the distribution was 46% Type 0, 3% Type 1, 46% Type 2, and 4% Type 3. These findings show that Modic Type 2 changes were the most prevalent form of vertebral endplate degeneration in this population, with no differences between groups.

## Discussion

In our study, patients with Pfirrmann grade 3 degeneration at the operated disc level reported significantly higher preoperative low back pain compared with those with grade 4 or 5. As expected for recent literature^5,15-18^, after endoscopic lumbar discectomy, both low back and leg pain, quantified as of the NRS score, and the ODI scores, improved in all patient groups. Despite leg pain having a similar behavior between the two groups, it is noticeable that the reduction in low back pain was higher in the Pfirrmann grade 3 group in all time frames of assessment (2, 14, 30, and 90 days after surgery) when compared with Pfirrmann grade 4 or 5 group. Our results suggest that surgical intervention is effective in alleviating leg pain and back pain in the early outcome, even in patients with early-stage IVD degeneration, who present higher back pain scores.

Recent literature reviews describe IVD because of inflammatory processes and biochemical changes^19^ that sensitize nociceptors in the annulus fibrosus (AF)^20^. Moreover, it is described^20,21^ that proinflammatory cytokines induce the expression of neurotrophins, further increasing innervation of the AF. Structural changes of the intervertebral disc have also been pointed as a mechanism promoting neural projections of nociceptive fibers into the inner-AF^20,22^. These processes work together to cause higher pain perception. As degeneration progresses (Pfirrmann grades 4 and 5)^12^, due to the chronic inflammation^23^, there's the possibility that cell's senescence^24,25^ result in a diminished cellular reaction and pain signaling. These results suggest that with loss of hydration and structural integrity, a more degenerated disk could be associated with less back pain.

The study found no significant differences in ODI scores between the groups at 30 and 90 days postoperatively. This indicates that, despite variations in pain relief, functional outcomes as measured by the ODI were comparable across different degrees of IVD. Papanastasiou et al^11^ found significantly different ODI scores after microdiscectomy when comparing Pfirrmann grade 4 or 5 group and Pfirrmann grade 1 or 2; however, these results are only described 48 months postoperatively.

The absence of a significant association between back pain and Modic Changes or different Pfirrmann grades of adjacent levels suggests that these factors may not substantially influence early postoperative outcomes. Previous research has shown mixed results concerning the clinical relevance of Modic Changes^10,26^. Some studies report associations between the presence or higher grades of Modic Changes with worse clinical outcomes, while others do not find a significant association. Modic changes may not be a critical determinant of short-term surgical outcomes.

Limitations of this study include the small sample size and the severe imbalance between groups, with only 10 patients classified as Pfirrmann grade 3 compared with 81 in the Pfirrmann 4 or 5 category. The study also does not account for other confounding variables such as body mass index, smoking status, occupation, physical activity levels, or comorbidities, all of which could affect surgical success. Finally, the classification of degenerative disc disease into only two groups (Pfirrmann 3 vs. Pfirrmann 4 or 5) may oversimplify a complex condition that progresses along a continuum, a more detailed stratification of Pfirrmann grades with a greater sample size could provide deeper insights into the relationship between IVD severity and surgical outcomes.

To improve the results provided by this study, future research should aim to increase sample size and, if possible, ensure a more balanced distribution between groups, opening the opportunity for a more detailed analysis.

## Conclusion

In this study, more pronounced preoperative back pain was associated with Pfirrmann grade 3 patients, by comparing with patients with Pfirrmann grades 4 and 5. Those patients showed a more substantial reduction in back pain after surgery, reaching NRS levels similar as those of patients with Pfirrmann 4 or 5 grades. These results can help decision-making and patient counseling regarding expected outcomes based on the severity of IVD.
